# Optimal layout of blasting holes in structural anisotropic coal seam

**DOI:** 10.1371/journal.pone.0218105

**Published:** 2019-06-18

**Authors:** Gaowei Yue, Minmin Li, Lu Wang, Weimin Liang

**Affiliations:** School of Civil Engineering, Henan Polytechnic University, Jiaozuo, China; Sichuan University, CHINA

## Abstract

Compared with the idealized isotropic coal seam, the effectively cracking distances of deep-hole pre-splitting blasting in different directions have significant difference in the structural anisotropy of coal seam. In this paper, based on the test results of coal mechanical parameters in different coring directions, the blasting crack distances in these coring directions of coal seam are theoretically calculated and analyzed, and then a new method of blasthole layout in coal seam is designed and the blasting crack effect is investigated according to the test data of gas drainage. The research results show that both compressive strength and tensile strength of the direction of perpendicular to bedding are larger than that in other directions, especially parallel to bedding. The blasting crack distances of calculation results are in great agreement with that of underground in-situ measurement, so the blasting crack zone caused by the differences of mechanical properties in different directions can be approximately treated as an ellipse. Comparing the conventional method of blasthole layout, the blasting crack effect with new method has greatly enhanced the methane concentration and gas drainage quantity. Pure methane quantities with the new method and conventional method are about 2.8 times and 1.67 times as large as that before blasting, and pure methane quantity with the new method is about 1.46 times that with the conventional method. Therefore, considering the anisotropic characteristics of coal seams, the reasonable blasthole layout plays an important role for enhancing gas drainage in low permeability and outburst working face.

## 1 Introduction

Coal mine methane (CMM) occurs naturally in coal seams as the adsorbed and free state, which is a kind of green energy with high burning value and little emissions after burning [1]. However, CMM is one of the threats for underground coal mine because the accumulated methane in the entry has the potential to trigger methane explosive, and high methane content in coal seams is one of the sufficient conditions for coal and gas outburst disaster [2–5]. In order to solve gas utilization and prevent gas disaster in coal mine, gas pre-extraction before coal seam mining is one of the important measures, however, the permeability of coal seams is low in most of high gas and outburst mines, and as the coal mining gets deeper and deeper, low-gas mines are gradually replaced by high-gas mines and the geological conditions are becoming increasingly complex. For example, over 70% of state-owned coal mines in China feature very low gas permeability making it immensely difficult to implement direct gas drainage, the essential measure of gas control. So conventional pre-pumping method is not able to solve these problems, which seriously restricts the safe production of coal mine and the development and utilization of coal mine methane [6].

Therefore, it is necessary to take effective measures to increase the permeability before coal seam mining, so as to improve gas extraction rate. At present, the most extensively study and application of coal seam permeability improvement measures mainly include drilling technology (large aperture drilling, dense drilling, cross drilling) [7–8], deep-hole blasting technology [9–11], hydraulic fracturing[12], etc[13–14], and the application of these measures has achieved the effect of increasing coal seam permeability. Currently deep-hole blasting technology is the most widely used [15–16], and many researchers have carried out a series of theoretical and practical studies on this technology in coal mines, especially in regard to the extended range of pre-splitting blasting. Almost all of the reports on the pre-splitting blasting in coal seam are supposed that the coal seam is homogeneous and isotropic, which causes that the shape of fracture range is a circle centered on the blasthole [17–19]. However, coal body is an anisotropy heterogeneous natural material containing both bedding and cleat structures. This unique feature makes coal showing anisotropy mechanical property [20–26], and the differences of the tensile and compressiion strength in different directions will cause different crack distances as the pre-splitting blasting is carried out in coal seam[17].

So the layout of blastholes and drainage holes should be optimized with considering the anisotropy mechanical property. In this work, firstly, the radius of pre-splitting blasting is deduced theoretically, and then the tests are carried out on mechanical parameters and effective blasting crack distances of coal seam. Finally, the layout of blastholes in coal seam is designed.

## 2 Theory of deep-hole blasting cracking

### 2.1 Load of deep-hole blasting

Hereon, the column charge in borehole is considered as coupling charge, the impact of the explosion was acted on the coal wall around blasthole. The explosive detonation pressure (*p*_0_) and the initial blast wave pressure (*p*) transmitted into coal can be obtained based on the acoustic approximation theory [27].

p=2ρcvcρcvc+ρevdp0 (1)

$$p_{0}=\frac{1}{1+\gamma }\rho_{e}v_{d}{}^{2}$$ (2)

Where, *ρ*_*c*_ and *ρ*_*e*_ are coal and explosive density respectively, kg/m^3^; *v*_*c*_ and *v*_*d*_ are the speed of sound and explosive detonation in coal respectively, m/s; *γ* is the expansion adiabatic index of detonation products, herein *γ* = 3.

Because the blast wave transmitting into coal body propagates outward and gradually attenuates, then it will become the stress wave. And the blast wave or the stress wave will cause the radial stress (*σ*_*r*_) and the tangential stress (*σ*_*θ*_) at any position (*r*) in coal body, which can be expressed as,

$$\sigma_{r}=p{\left(r/r_{b}\right)}^{\text{-}\alpha }$$ (3)

$$\sigma_{\theta }=-b\sigma_{r}$$ (4)

Where, *r* is the distance from any point to the center of the charge, m; *r*_*b*_ is the radius of the blasthole, m. The lateral pressure coefficient (*b*) and the propagation attenuation index (*α*) of blast/stress wave can be expressed as follows respectively:

$$b=\mu_{d}/(1-\mu_{d})$$ (5)

α=2±b (6)

Where, *μ*_*d*_ is dynamic Poisson’s ratio of coal body, and in engineering application, *μ*_*d*_ is generally considered as *μ*_*d*_ = 0.8*μ*, here *μ* is the static Poisson’s ratio. The plus or minus signs in the Eq (6) corresponds to blast wave zone and stress wave zone respectively.

### 2.2 Stress state and failure criterion of coal body

In practical engineering, coal body is generally in three-dimensional stress state. So the stress (*σ*_*i*_) at any point in coal body can be expressed as,

$$\sigma_{i}=\frac{1}{\sqrt{2}}{\left[{\left(\sigma_{r}\text{-}\sigma_{\theta }\right)}^{2}\text{+}{\left(\sigma_{\theta }\text{-}\sigma_{z}\right)}^{2}{\left(\sigma_{z}\text{-}\sigma_{r}\right)}^{2}\right]}^{\frac{1}{2}}$$ (7)

Where, *σ*_*z*_ is axial stress, MPa.

If the blasting cracking of coal body is regarded as a plane strain problem along radial direction, then *σ*_*z*_ can be expressed as,

$$\sigma_{z}=\mu_{d}(\sigma_{r}+\sigma_{\theta })=\mu_{d}(1-b)\sigma_{r}$$ (8)

Substituting Eq (8) into Eq (7), then *σ*_*i*_ can be changed as,

$$\sigma_{i}=\frac{1}{\sqrt{2}}\sigma_{r}{\left[{\left(1+b\right)}^{2}-2\mu_{d}{\left(1-b\right)}^{2}(1-\mu_{d})+(1+b^{2})\right]}^{\frac{1}{2}}$$ (9)

During the process of the pre-splitting blasting in coal seam, the coal body around the blasthole is pressed by blast wave to produce a crushing zone. And the coal body at some distance is tensed by the stress wave to radial crack zone. Coal body is brittle material, and its uniaxial compressive strength is several times (3~10 times) than the uniaxial tensile strength, so even further from the blasting point, the coal body will still be cracked by the stress wave.

According to Mises criterion, if the stress *σ*_*i*_ of coal body satisfies the following Eq (10) in crushing zone, the coal body will be destroyed,

$$\sigma_{i}\geq \sigma_{cd}$$ (10)

Where, *σ*_*cd*_ is dynamic compressive strength of coal, MPa.

At the boundary of crushing zone, the equivalent stress intensity (*σ*_*i*_) in coal decreases to the uniaxial dynamic compressive strength (*σ*_*cd*_) of coal.

Under the impact load, different coal bodies have different sensitivity to strain. Generally, the dynamic compressive strength of coal increases with increasing loading strain rate. The results show that the relationship between dynamic compressive strength and static compressive strength of coal body can be approximated by Eq (11)[28–29],

$$\sigma_{cd}=\sigma_{c}\sqrt[{3}]{\dot{\varepsilon }}$$ (11)

Where, *σ*_*c*_ is the uniaxial static compressive strength of coal, MPa; $\dot{\varepsilon }$ is the strain rate, s^-1^. During the engineering blasting, $\dot{\varepsilon }$ is bigger in crushing zone, which is about between 10^2^ and 10^4^ s^-1^, and it is smaller outside the compression zone, which is less than 10^3^ s^-1^.

In radial crack zone, the coal body is tangential stretching failure, It can be seen from Mises criterion,

$$\sigma_{i}\geq \sigma_{td}$$ (12)

Where, *σ*_*td*_ is the uniaxial dynamic tensile strength of coal, MPa. However, the dynamic tensile strength of coal body hardly varies with the loading strain rate, i.e. *σ*_*td*_ = *σ*_*t*_, so Eq (13) is applied as the coal crack of the stress wave,

$$\sigma_{i}\geq \sigma_{t}$$ (13)

Where, *σ*_*t*_ is the uniaxial static tensile strength of coal, MPa.

### 2.3 Effective distance of blasting crack

After blasting, the coal body around the borehole is crushed by the blast wave, and forms a crushing zone. The radius of crushing zone can be obtained from Eqs (1)~(3), (9) and (10):

$$R_{C}={\left(\frac{AB}{\sqrt{2}\sigma_{cd}}\right)}^{1/\alpha }r_{b}$$ (14)

Where,A=2ρcvcρcvc+ρevdρevd21+γ, $B={\left[{\left(1+b\right)}^{2}+(1+b^{2})-2\mu_{d}(1-\mu_{d}){\left(1-b\right)}^{2}\right]}^{\frac{1}{2}}$, *α* is the attenuation index of the blast wave, *α =* 2+*b*.

At the interface of the crushing zone and the crack zone, the radial stress (*σ*_*R*_) in the coal body can be expressed as Eq (15) according to Eqs (9) and (10),

$$\sigma_{R}=\sigma_{r}|{}_{r=R_{c}}\frac{\sqrt{2}\sigma_{cd}}{B}$$ (15)

Outside the crushing zone, the blast wave continues to propagate outward in the form of stress wave. In order to distinguish the attenuation index of the blast wave, the attenuation index of the stress wave is expressed with *β*, and *β =* 2-*b*. The radius of the crack zone is obtained by Eqs (1)~(3), (9) and (13),

$$R_{F}={\left(\frac{\sigma_{R}B}{\sqrt{2}\sigma_{td}}\right)}^{1/\beta }R_{C}$$ (16)

Substitute Eqs (4) and (5) into Eq (16), the radius of the crack zone changes as,

$$R_{F}={\left(\frac{\sigma_{cd}}{\sigma_{td}}\right)}^{1/\beta }{\left(\frac{AB}{\sqrt{2}\sigma_{cd}}\right)}^{1/\alpha }r_{b}$$ (17)

## 3 The blasting crack distance in structurally anisotropic coal seam

### 3.1 Tests of mechanical parameters

#### 1) Specimen preparation

**Fig 1:**
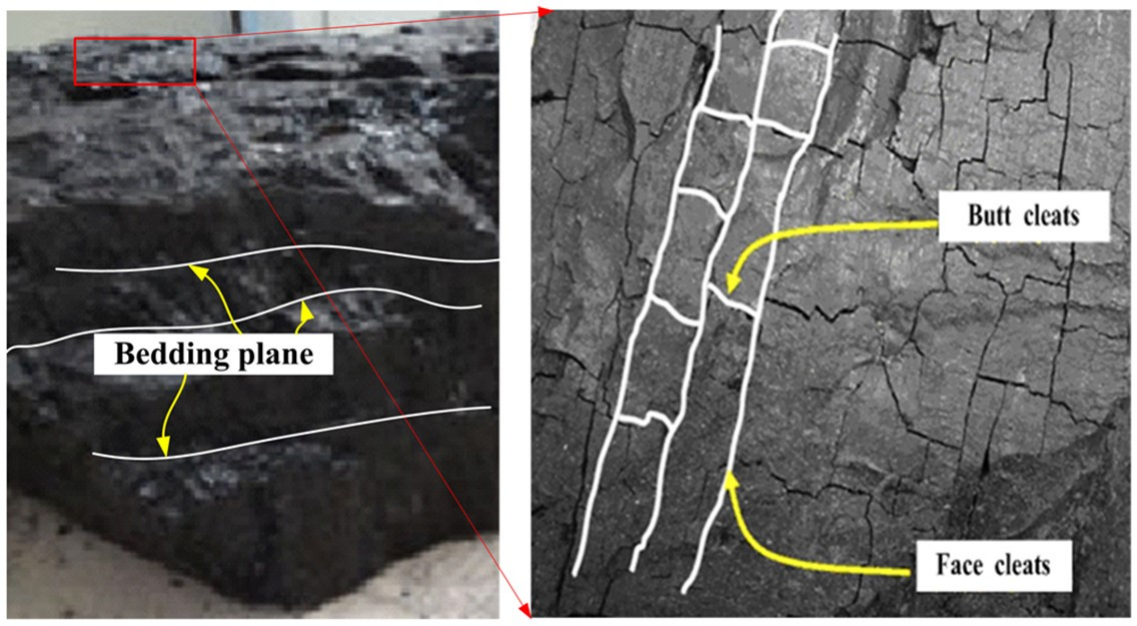
Characteristics of bedding and joints of coal.

Coal is a complex porous media, which is composed of coal matrix, porosity and cracks. After a long geologic age, coal seam has obvious bedding characteristics due to the differences of coal material composition, particle size, cements and structure (as shown in Fig 1). The bedding is the most extensive structure in coal seam basin, and the coal bedding plane has clear hierarchy [30–32]. Some cleats have developed in vertical bedding planes direction, and cleats are approximate parallel and their distribution is not continuous. In coal seem there are two groups of perpendicular cleats, which are called face cleat and butt cleat respectively according to their form and intersected relations [33]. The bedding planes and cleats destroy the continuity and integrity of coal body, which has decisive influence for mechanical property in different directions [32].

**Fig 2:**
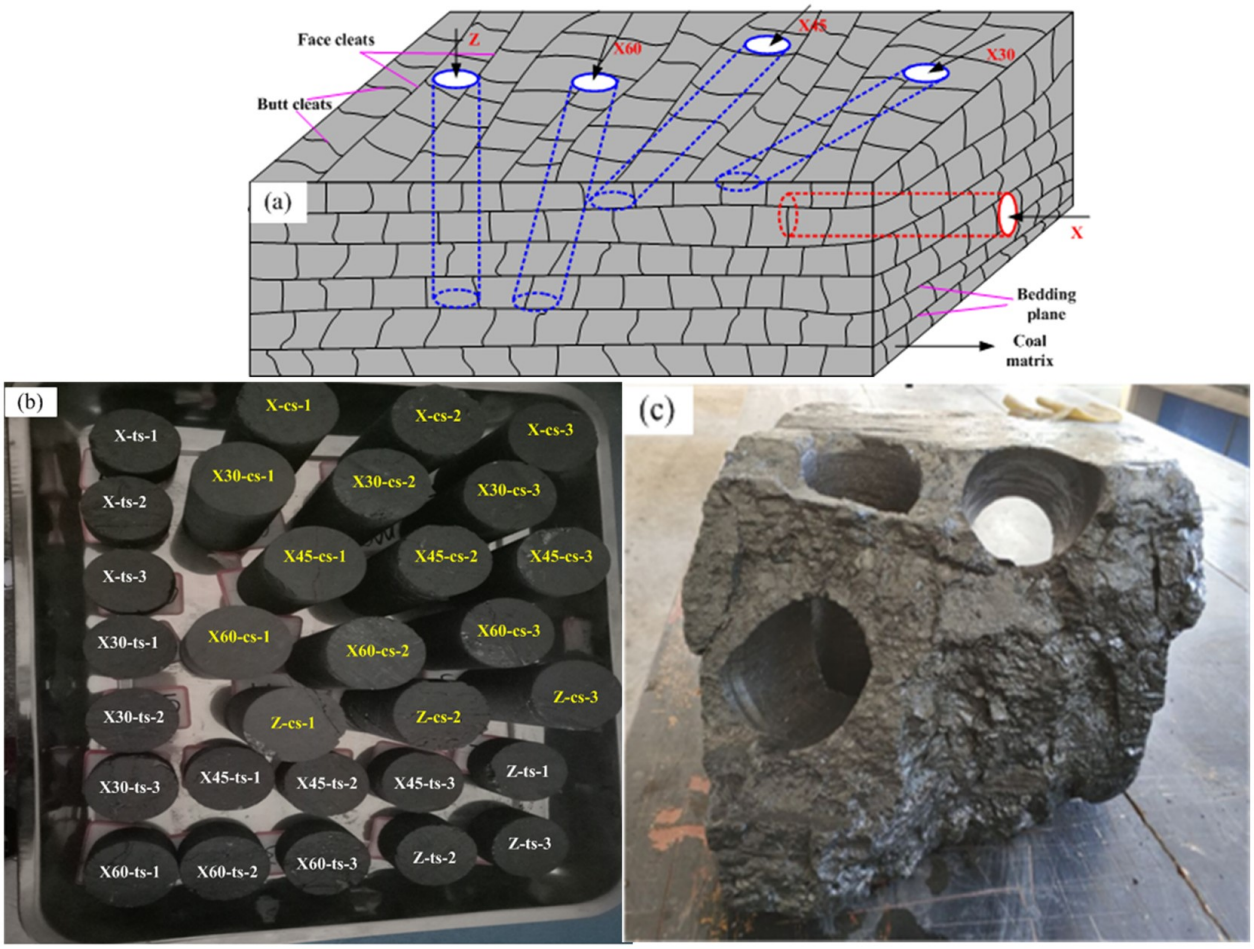
Coal sample preparation (a) Core direction (b) Coal sample (c) The coal form after multi-direction sample.

Coal blocks are obtained from Second-1 coal seam in Jiulishan Coal Mine, Jiaozuo, China. The coal seam is so hard that the coefficient of hardness (*f*) is 1.34. The bedding surface of coal blocks is obvious, and there are natural butt cleats and face cleats. Coal samples are prepared in five directions of coal blocks as shown in Fig 2a; sample X: the X direction is parallel to bedding plane in the face cleat direction; sample X30, sample X45 and sample X60: the X30, X45 and X60 directions are 30°, 45° and 60° to X direction respectively; sample Z: the Z direction is perpendicular to the bedding plane. Coal bodies for sampling are shown in Fig 2c. After coal samples are taken out, their upper and lower end faces are polished to be smooth and parallel using cutting machine. The prepared coal specimens with *Φ* 50 × 100mm and *Φ*50×30mm are shown in Fig 2(b), and the serial numbers of coal specimens with “cs” and “ct” represent that the coal specimens are for compression strength test and tensile strength test respectively.

#### 2) Test results and analysis of mechanical parameters

**Fig 3:**
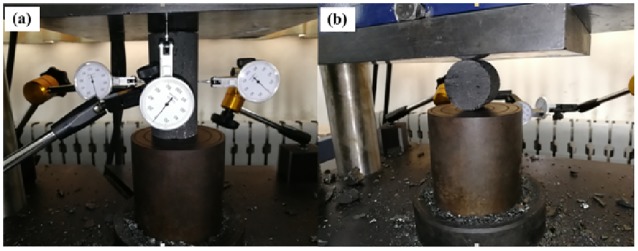
Mechanical testing (a) Compression test (b) Tensile test.

Electronic universal testing machine is adopted to carry out the uniaxial compression test and Brazilian splitting test (tensile test), as shown in Fig 3. And the radial changes of coal specimens are measured with dial indicators during the uniaxial compression test. Before test, diameter and length of each coal specimen are measured five times at five different positions with vernier caliper. Samples of each type should be tested at least for three times (there are some spare coal samples). The loading speed of the tests is 0.2kN/s.

**Fig 4:**
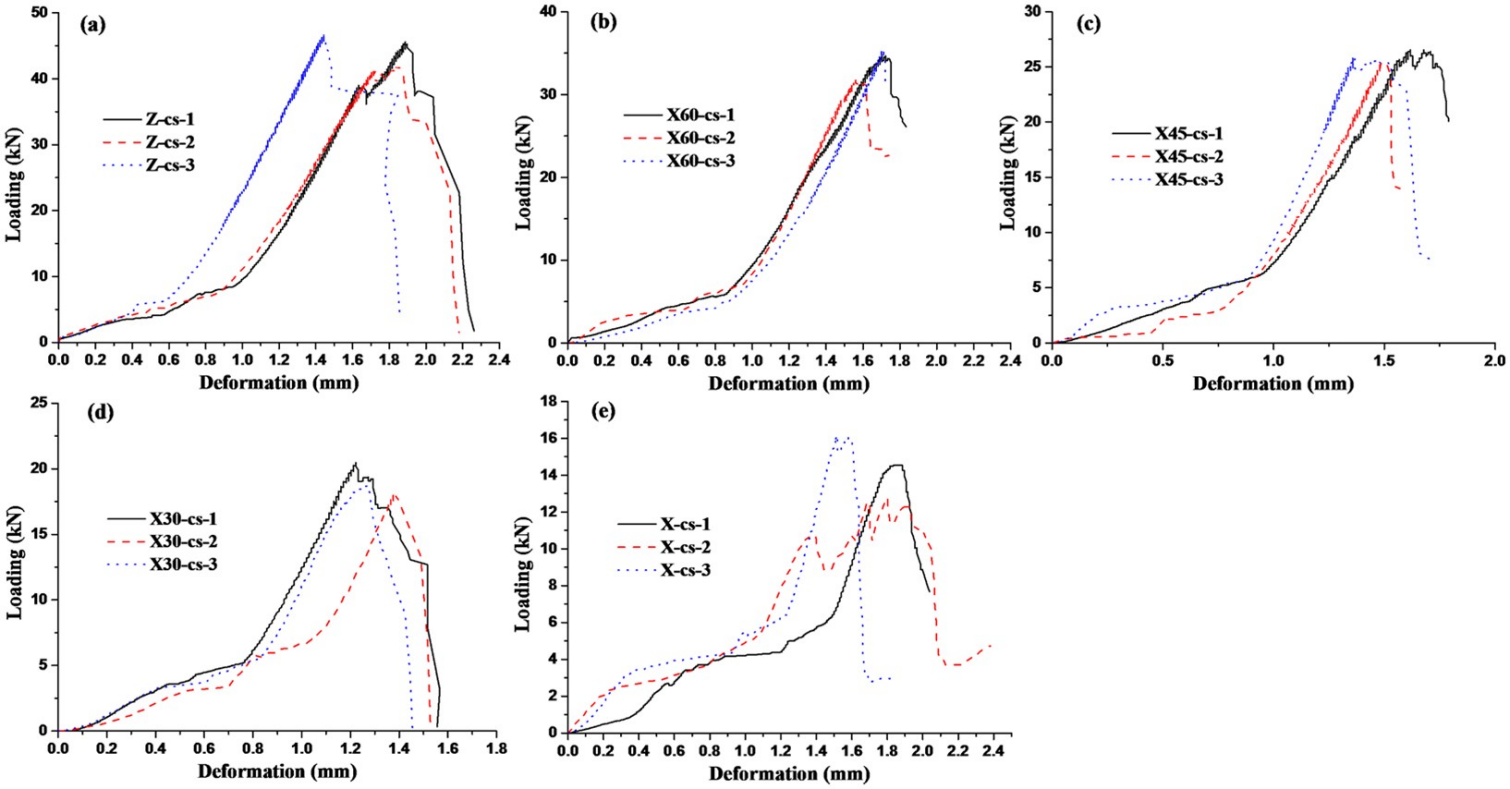
Load-deformation curve of uniaxial compressive strength test.

**Fig 5:**
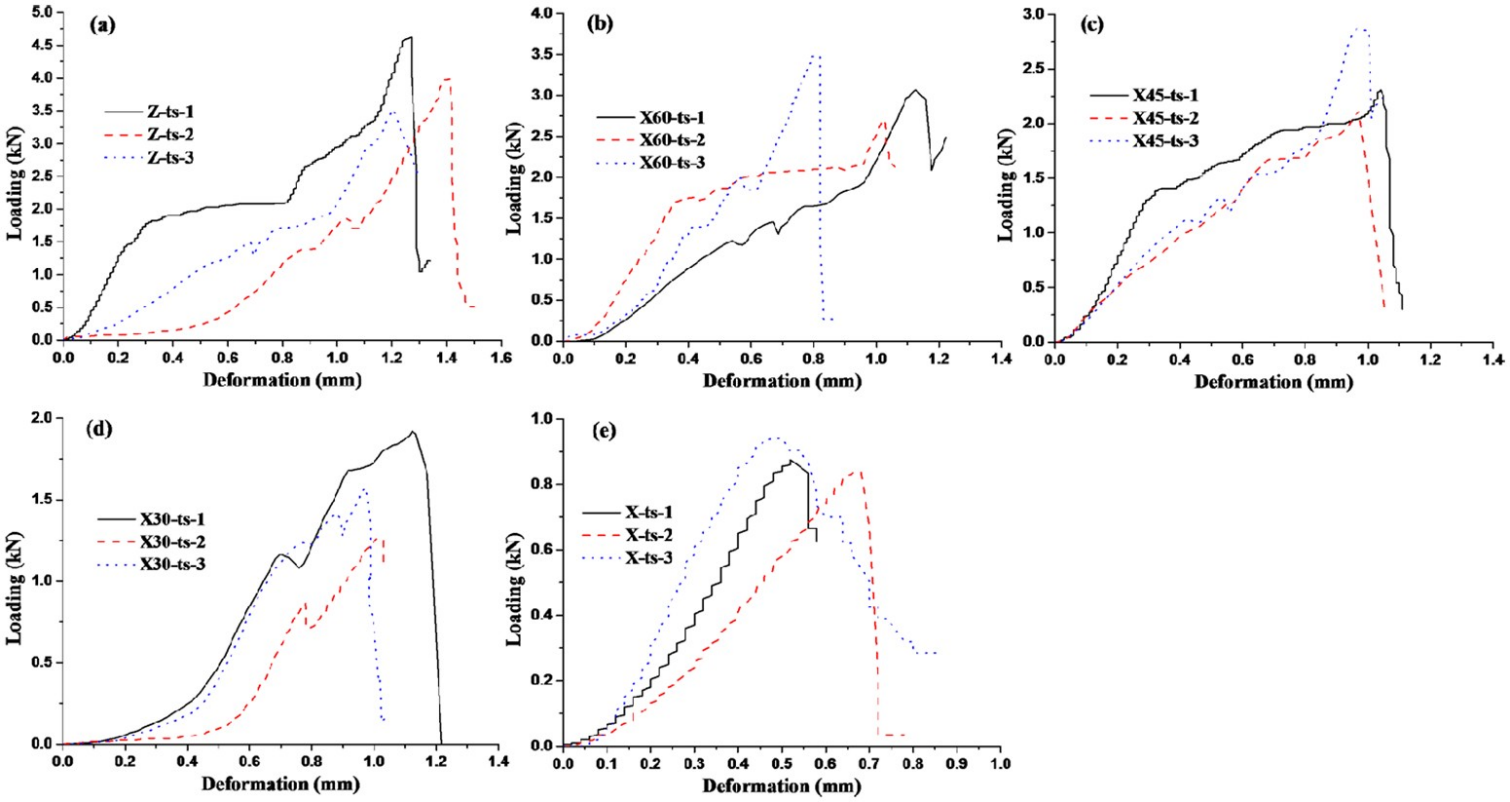
Load-deformation curve of uniaxial tensile strength test.

The deformation curves of coal samples with loading are shown in Figs 4 and 5, which are the uniaxial compression tests and Brazilian splitting tests (tensile test) respectively. It can be seen from Figs 4 and 5 that in the initial stage of loading, the force is smaller, but deformation is larger, which indicates that coal body is re-compacted. Then the force increases sharply and reaches maximum value with smaller deformation, and the force drops rapidly after the maximum value that fully illuminates that coal sample has been destroyed and coal is brittle materials. According the measured data, Poisson ratio, the compressive and tensile strength can be calculated as shown in Table 1. Both compressive strength and tensile strength are the largest in Z direction and are the smallest in the X direction. Moreover, the compressive strength and tensile strength decrease with included angle between core direction and X direction decreasing. Meanwhile, the compressive strength is more than 13 times of the tensile strength on the same direction. Poisson ratio of coal is about 0.3.

**Table 1:** Test results of compressive and tensile strength.

Serial number	compressive strength (MPa)	Mean value	Poisson ratio	Mean value	Serial number	tensile strength (MPa)	Mean value
Z-cs-1	23.21	22.78	0.316	0.32	Z-ts-1	1.96	1.715
Z-cs-2	21.29		0.332		Z-ts-2	1.695	
Z-cs-3	23.86		0.312		Z-ts-3	1.495	
X60-cs-1	17.66	17.36	0.287	0.291	X60-ts-1	1.3	1.305
X60-cs-2	16.55		0.274		X60-ts-2	1.145	
X60-cs-3	17.88		0.312		X60-ts-3	1.475	
X45-cs-1	13.53	13.13	0.272	0.282	X45-ts-1	0.96	0.975
X45-cs-2	12.95		0.321		X45-ts-2	0.85	
X45-cs-3	12.92		0.253		X45-ts-3	1.12	
X30-cs-1	10.43	9.55	0.292	0.278	X30-ts-1	0.87	0.71
X30-cs-2	8.99		0.262		X30-ts-2	0.555	
X30-cs-3	9.22		0.28		X30-ts-3	0.71	
X-cs-1	7.40	7.35	0.303	0.302	X-ts-1	0.465	0.475
X-cs-2	6.49		0.311		X-ts-2	0.555	
X-cs-3	8.17		0.292		X-ts-3	0.41	

### 3.2 Calculation results and analysis of crack distance

**Fig 6:**
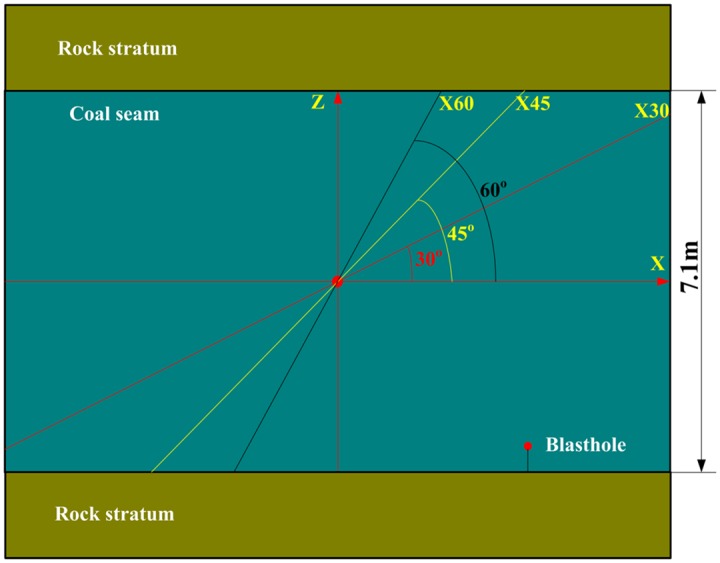
Calculation model of blasting crack.

The values of the explosive property parameters are as follows: the density is 1130 kg/m^3^, detonation velocity is 3500 m/s, and the sound velocity in coal body is 2200 m/s [34]. The radius of blasthole is 45 mm, and the density of coal is 1450 kg/m^3^. According to the coring direction (Fig 2a), a simple model of blasting cracking in coal seam is established as shown in Fig 6. Then all the parameters including the compressive and tensile strength are substituted into Eqs (14) and (16), the distances of the crushing and crack distances of coal seam in different directions are calculated and shown in Table 2.

**Table 2:** The effective distances of the crushing and crack zones.

Direction	*σ*_*c*_ (MPa)	*σ*_*t*_ (MPa)	*R*_*C*_/*r*_*b*_	*R*_*F*_/*r*_*b*_	*R*_*C*_ (m)	*R*_*F*_ (m)
Z	22.78	1.715	3.908	55.518	0.176	2.615
X60	17.36	1.305	4.404	62.096	0.198	2.794
X45	13.13	0.975	4.997	69.643	0.225	3.134
X30	9.55	0.71	5.757	79.522	0.259	3.578
X	7.35	0.475	6.346	100.25	0.286	4.511

**Fig 7:**
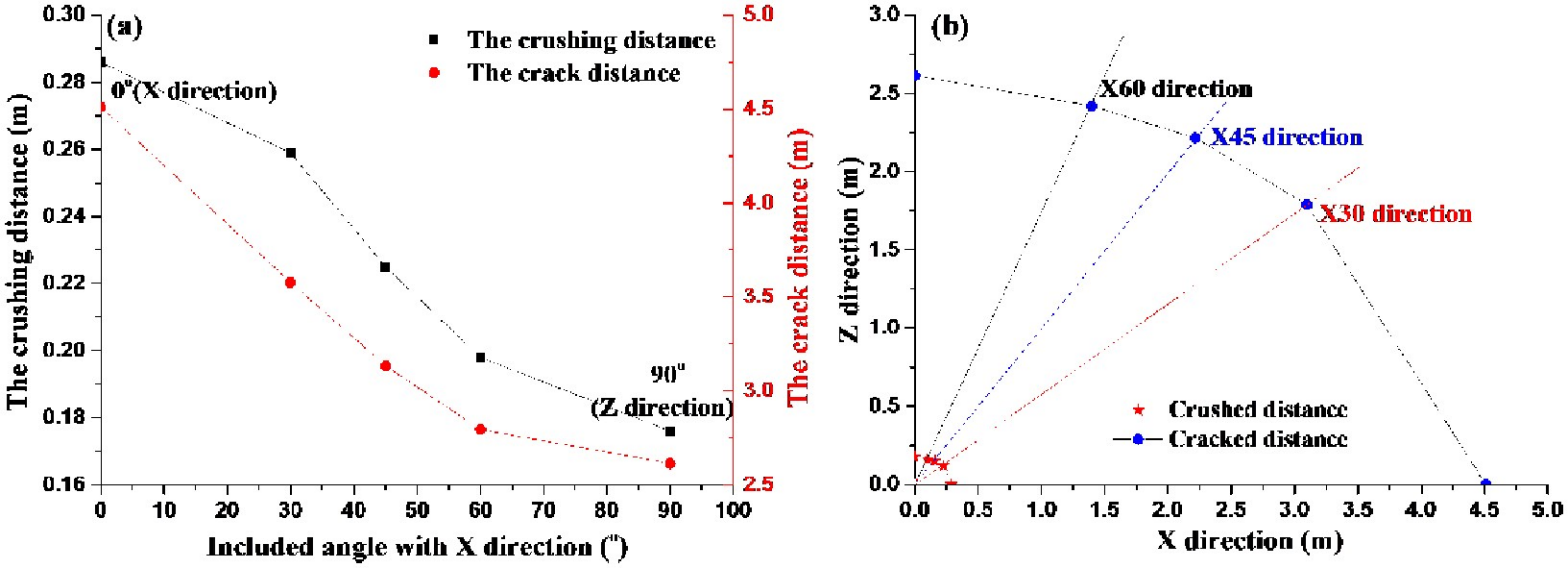
The crushing and crack distances of blasting (a) The crushing and crack distances in different direction (b) The crushing and crack zones.

In Table 2, the distances of the crushing and cracking zones increase with the compressive and tensile strength decreasing, which is due to he mechanical property differences of coal body in different directions, as shown in Fig 7a, and in XZ rectangular coordinate system, the crushing and cracking zones (a quarter) are shown in Fig 7b. From Fig 7, it can be seen that the smaller the included angle with X direction is, the smaller compressive and tensile strength are, but the larger the crushing and cracking distances are. For example, the crushing distance in Z, X45, X directions are 0.176m, 0.225m, 0.286m respectively; the crack distance in Z, X45, X directions are 2.615m, 3.134m, 4.511m respectively, which indicates that the crack distances of coal in different directions are obviously different during blasting. And the crack distances in X direction is about 1.7 times as that in Z direction; the crushing distances in X direction is about 1.6 times as that in Z direction. Moreover, the crushing distance in coal seam is 3.5~7 times of charge radius, and the crack distance is 50~100 times of charge radius.

## 4 Blasthole design in coal seam

Before designing blasthole layout in coal seam, it needs to investigate correctness of calculation results. So field test is arranged at No. 16 mining area of Second-1 coal seam in Jiulishan coal mine, Jiaozuo, China. The thickness of coal seam is between 6.1m and 8.1m, and the average thickness is 7.1m. Gas extraction is carried out after deep-hole blasting.

### 4.1 Investigation on effective crack distance of blasting in coal seam

**Fig 8:**
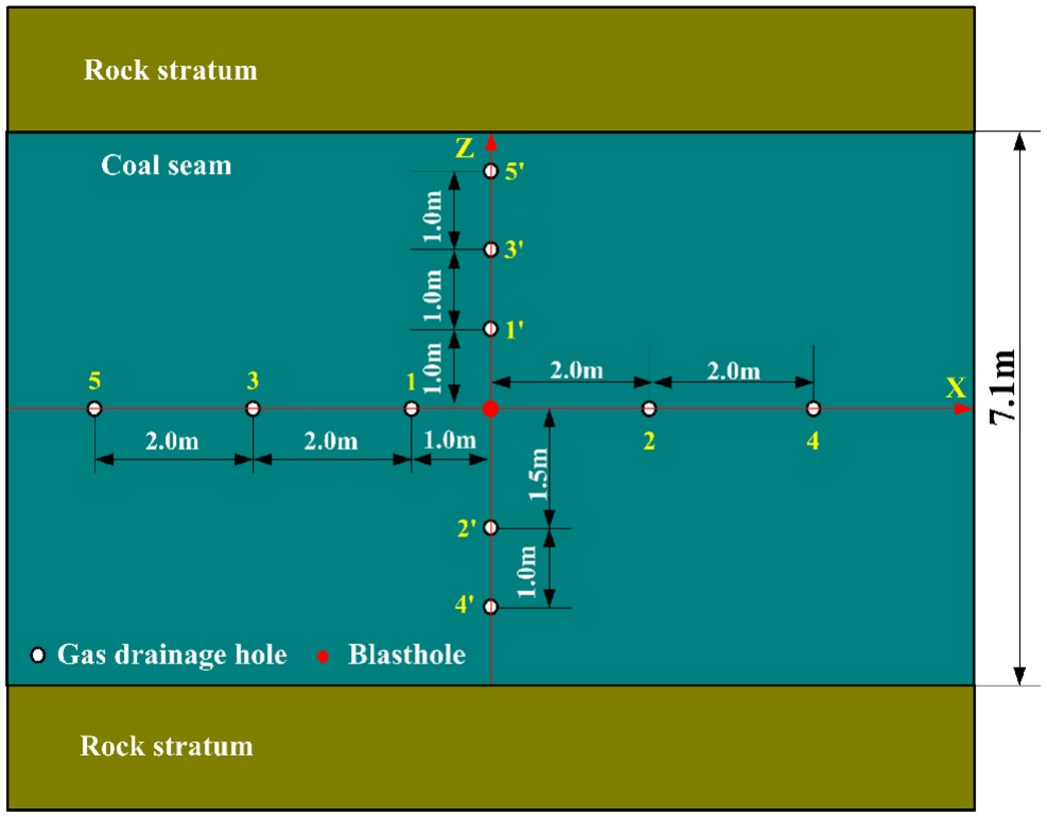
Hole layout for blasting effect investigation.

In order to investigate the effect of the blast cracking in different directions, blasthole and gas extraction holes were designed as shown in Fig 8. The holes of No.1~No.5 are in parallel to bedding direction, and the holes of No.1'~No.5' are in perpendicular to bedding direction.

**Fig 9:**
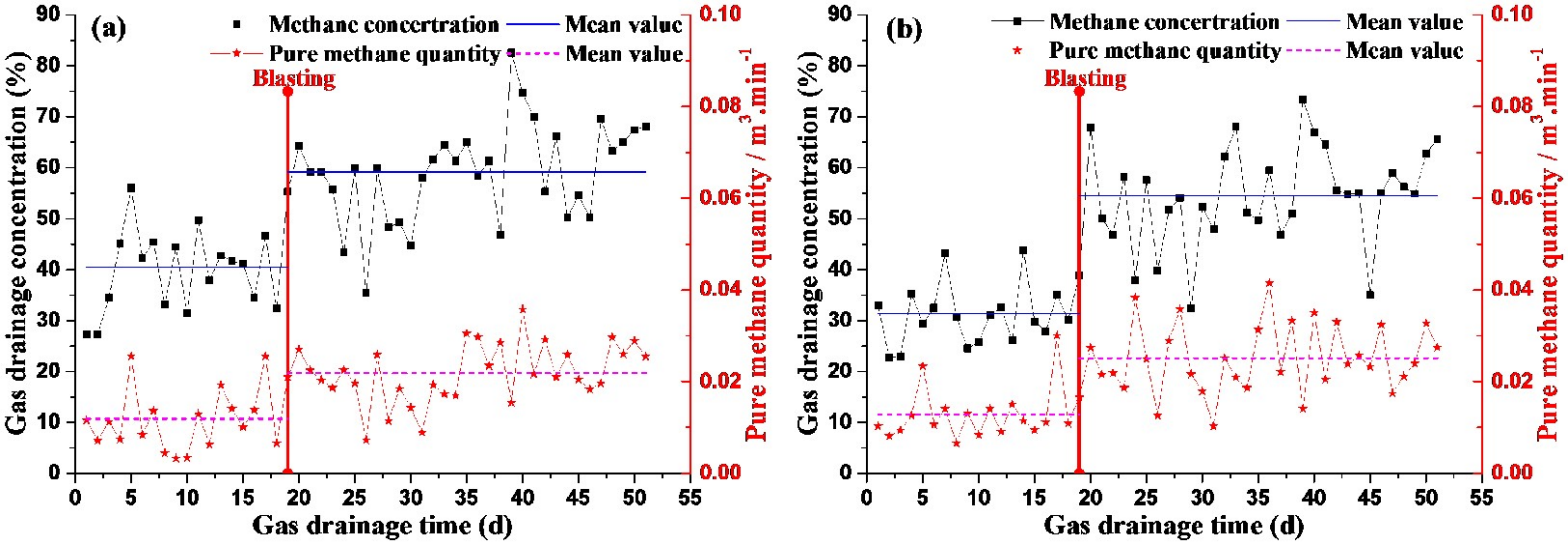
Curve diagrams of gas drainage concentration and pure methane quantity (a) No.2 gas drainage hole (b) No.2’ gas drainage hole.

Before blasting, gas drainage has continued for 19 days, and the methane concentration and pure methane quantity of each gas drainage hole are measured and recorded, so their mean values of each gas drainage hoe in 19 days can be obtained. After blasting, the same job is to continue measuring and recording the methane concentration and pure methane quantity of each gas drainage hole until the fifty-first day. Herein, taking gas drainage holes of No.2 and No.2' as examples, the methane concentration (daily mean value) and pure methane quantity (daily mean value) are recorded as shown in Fig 9.

Fig 9 shows that before blasting, both the methane concentration and pure methane quantity are smaller than that after blasting. For gas drainage from the No.2 hole, the mean value of methane concentration and pure methane quantity with 19 days before blasting are 40.46% and 0.0476m^3^/min respectively, however, the mean value of methane concentration and pure methane quantity with 32 days after blasting are 59.16% and 0.0876 m^3^/min respectively, which are 1.46 times and 1.84 times as big as that before blasting respectively. For gas drainage from the No.2′ hole, the mean value of methane concentration and pure methane quantity with 19 days before blasting are 31.35% and 0.0515 m^3^/min respectively, however, the mean value of methane concentration and pure methane quantity with 32 days after blasting are 54.5% and 0.1005 m^3^/min respectively, which are 1.74 times and 1.95 times bigger than before blasting respectively.

**Fig 10:**
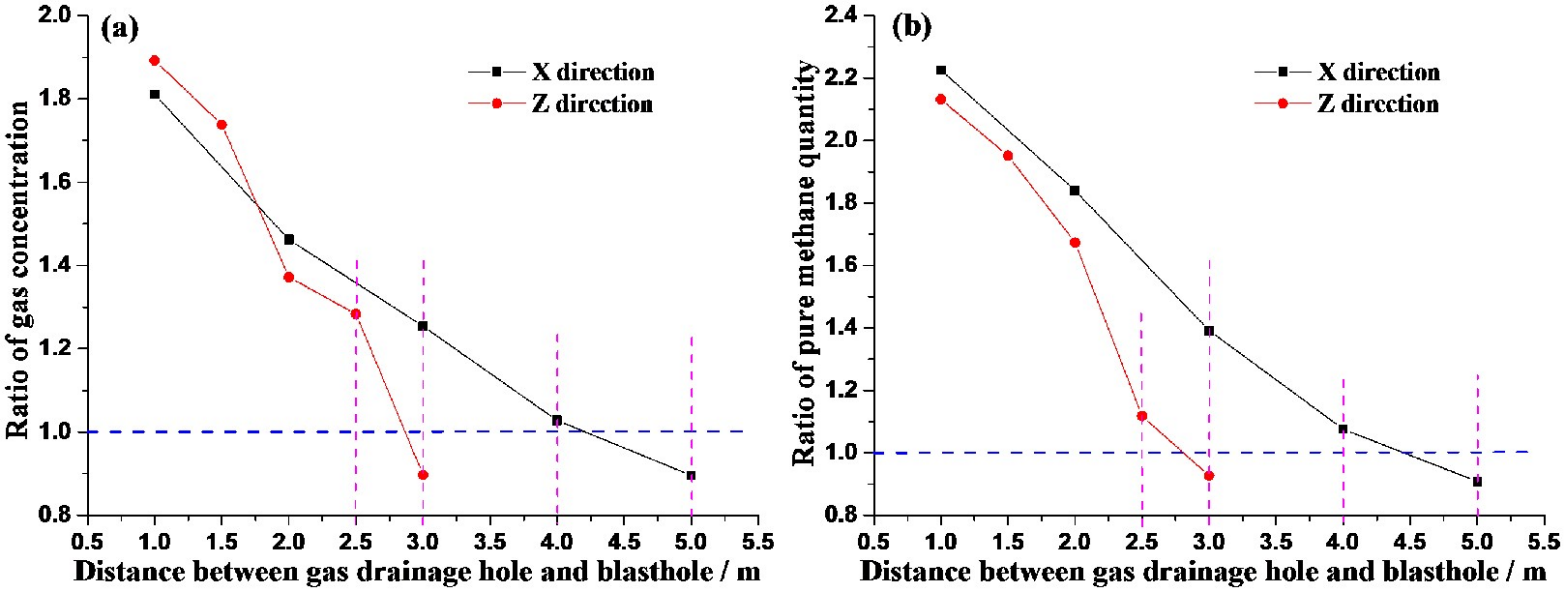
Ratio after and before blasting with the different distances between gas drainage hole and blasthole (a) Methane concentration (b) Pure methane quantity.

Using the same method as Fig 9, for gas drainage of each hole, the mean value of the methane concentration and pure methane quantity can be obtained as shown in Table 3, so it is easy to get their ratios after and before blasting. Fig 10 shows the relationship of the ratios and the distances between gas drainage hole and blasthole. It can be seen from Fig 10, the closer gas drainage hole is to blasthole, the bigger the ratio is, which indicates that after blasting, the closer gas drainage hole is to blasthole, the more obviously the methane concentration and pure methane quantity enhance. This is because that blasting has effectively changes the pore and fissure structure of coal body, which improves gas drainage quantity and gas extraction rate. However, in different directions (X and Z), the effective influence distances caused by explosion have significant differences. Herein, the ratio 1.0 is as a criterion value to judge whether the gas drainage hole is affected by blasting. From Fig 10, the ratios of methane concentration and pure methane quantity show that in X direction the effective influence distance is between 4.0m and 5.0m, and in Z direction the effective influence distance is between 2.5 m and 3.0m.

**Table 3:** The measurements of blasting cracking effect.

Comparison Direction	Mean value of gas drainage concentration (%)				Mean value of pure methane quantity (m^3^/min)		
	Number of hole	Before blasting	After blasting	Ratio after and before blasting	Before blasting	After blasting	Ratio after and before blasting
X	1	41.44	75.04	1.81	0.0128	0.0286	2.23
	2	40.46	59.16	1.46	0.0119	0.0219	1.84
	3	38.51	48.29	1.25	0.0127	0.0177	1.39
	4	38.03	39.08	1.03	0.0122	0.0131	1.08
	5	33.53	30.03	0.89	0.0104	0.0094	0.91
Z	1’	38.9	70.38	1.89	0.0112	0.0239	2.13
	2’	31.35	54.5	1.74	0.0129	0.0251	1.95
	3’	33.08	48.13	1.45	0.0115	0.0193	1.67
	4’	32.23	36.37	1.13	0.01168	0.0131	1.12
	5’	22.28	19.98	0.90	0.00818	0.0076	0.93

Comparing the calculation results in section 3 with the field test results, the crack distance of blasting is calculated as 4.511m in X direction, which is just between 4.0m and 5.0m of field test. And the crack distance of blasting is calculated as 2.615m in Z direction, which is just between 2.5m and 3.0m of field test. The calculated results are fine in agreement with the experimental results. Furthermore, both the calculation results and the field test results show that the effective influence distances of blasting in coal seam have obvious differences in different directions. And these differences are very important to be considered in blasthole layout in coal seam.

### 4.2 Blasthole layout in coal seam

In order to design a blasthole layout for a mining panel, the conflict must be reasonably addressed to minimize the total blasthole numbers (cost) and maximize the blasting crack zone for enhancing the gas drainage efficiency of coal production. As mentioned earlier, the more numbers of the blastholes, the closer space between blastholes and the better the blasting crack effect, the higher the gas drainage efficiency. However, the less numbers of the blastholes would not achieve good cracking effect, which usually causes the longer drainage time and leads to a low coal mining efficiency, and it finally results in a low mining profit. It can be imagined that if the effective blast crack zone is obtained, the reasonable layout of blastholes can be designed to achieve best cracking effect by balancing borehole drilling cost and drainage duration.

**Fig 11:**
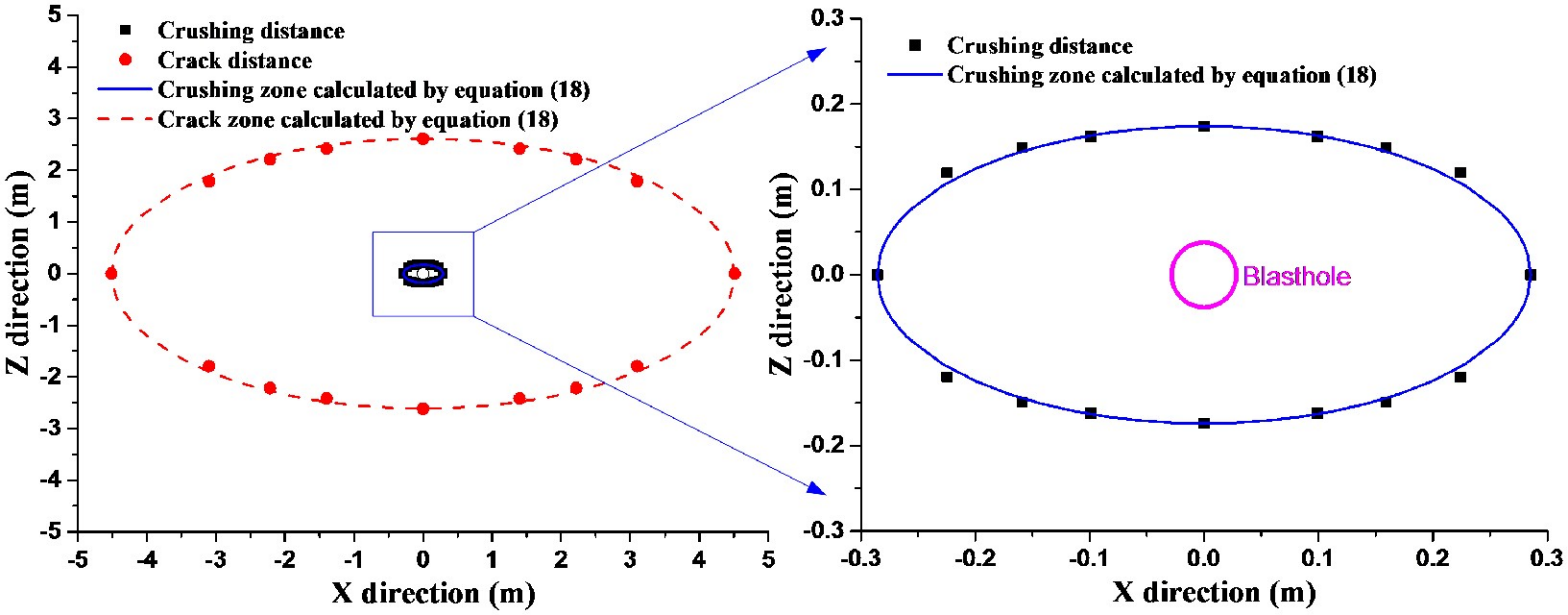
Blasting crushing-crack distance of coal seam.

Assuming that the crushing zone and cracking zone in coal body are symmetrical with X axis and Z axis (as shown in Figs 6 and 8), then the crushing and cracking distances in different directions shown in Fig 7b can be changed as in Fig 11. It can be seen from Fig 11 the blasting crushing and cracking zones are approximate ellipse, which are not circular calculated by conventional isotropous model.

When the cracking zones can be treated as an ellipse phenomenologically, the cracking distances in X and Z direction are *r*_*l*_ and *r*_*s*_, which are treated as the major and minor axis of the ellipse, the effective cracking distance to the blasthole center (*x*, *z*) follows Eq (18),

$$\frac{x^{2}}{r_{l}{}^{2}}+\frac{z^{2}}{r_{s}{}^{2}}=1$$ (18)

In order to validate the phenomenological idea of the ellipse-shape area, Eq (18) is used to fit simulation results presented in Fig 11. It is clearly that the proposed model (Eq (18)) fits simulation results very well as shown in Fig 11. This, on the other hand, supports that the effective crack zone of the blasting in an anisotropic coal seams is an ellipse.

**Fig 12:**
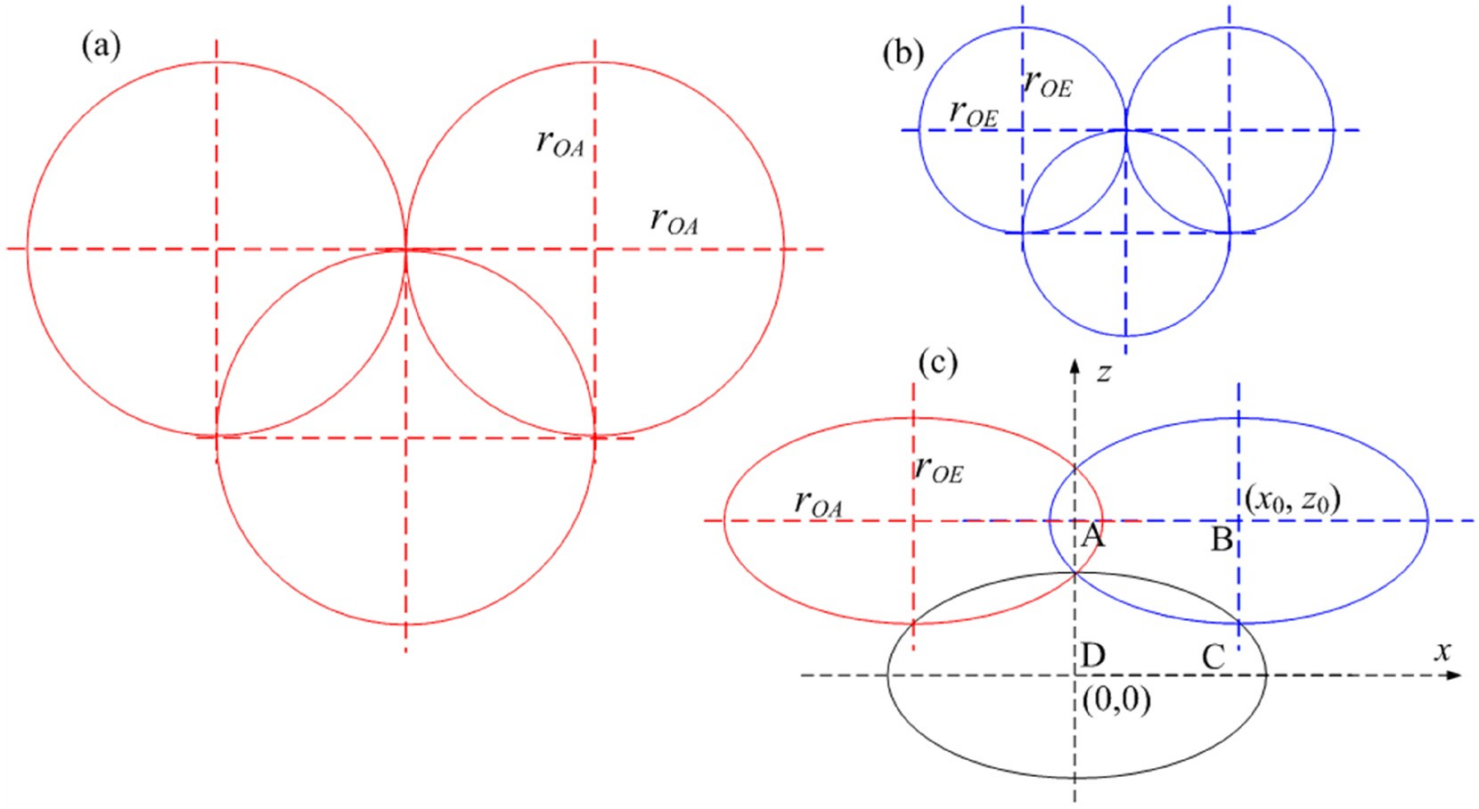
Simplified blasthole layout schematic diagram.

In order to ensure the outburst-prone risk of coal seam is fully eliminated, the uncracked area of blasting among each blasthole must be eliminated though a proper blasthole layout for efficient gas drainage. This means the coal seam must be fully covered the crack area of blasting. If the local area in the coal seam is out of the effective cracking area of blasting, the permeability is so low that it is hard to determine whether this area is out of outburst-prone risk within the gas pre-drainage time. For a specific mining panel, this problem can be simplified as the following mathematical problem: how to fully cover a rectangular panel area using minimum number of a same ellipse. Considering the symmetric feature in the problem, a simplified diagram is shown in Fig 12.

Assuming the coordinate of the first blasthole is at (0, 0), the effective cracking area of the blasthole is an ellipse with major axial (*r*_*l*_) and minor axial (*r*_*s*_) as shown in Fig 12. One of the nearest neighbor blastholes is at (*x*_0_, *z*_0_). In order to get the best blasthole layout, the intersection rectangular area (ABCD) between the effective cracking area of blasthole (0,0) and (*x*_0_, *z*_0_) must be the maximum. This means the product of (*x*_0_*z*_0_) is the maximum. In order to meet this criteria, the point (0, *r*_*s*_) must in the ellipse, $\frac{{\left(x-x_{0}\right)}^{2}}{r_{l}{}^{2}}+\frac{{\left(z-z_{0}\right)}^{2}}{r_{s}{}^{2}}=1$ and 0 < *x*_0_ < *r*_*l*_, r_s_ < *z*_0_ < 2*r*_*s*_. This criterion can be rewritten as Eq (19),

$$\frac{x_{0}{}^{2}}{r_{l}{}^{2}}+\frac{{\left(r_{s}-z_{0}\right)}^{2}}{r_{s}{}^{2}}=1$$ (19)

When the area (ABCD) is the maximum, the product of (*x*_0_*z*_0_) must be the maximum and the (*x*_0_*z*_0_)^2^ will also be the maximum. The (*x*_0_*z*_0_)^2^ term can be expanded as Eq (20),

$${\left(x_{0}z_{0}\right)}^{2}=r_{l}{}^{2}[1-\frac{{\left(r_{s}-z_{0}\right)}^{2}}{r_{s}{}^{2}}]z_{0}{}^{2}=\frac{r_{l}{}^{2}}{r_{s}{}^{2}}[r_{s}{}^{2}-{\left(r_{s}-z_{0}\right)}^{2}]z_{0}{}^{2}=\frac{r_{l}{}^{2}}{r_{s}{}^{2}}[2r_{s}-z_{0}]z_{0}{}^{3}$$ (20)

From Table 2, *r*_*l*_ = 4.511m and *r*_*s*_ = 2.615m can be obtained easily. So substituting *r*_*l*_ and *r*_*s*_ into Eq (20), the (*x*_0_*z*_0_)^2^ can calculated with different assignments of *z*_0_, and when *z*_0_ is 3.9225m, (*x*_0_*z*_0_)^2^ reaches the maximum value. So *x*_0_ calculated by Eq (19) is 3.9066m. For the convenience of engineering design, the values of *x*_0_ and *z*_0_ are determined as 4.0 m and 4.0 m, respectively.

**Fig 13:**
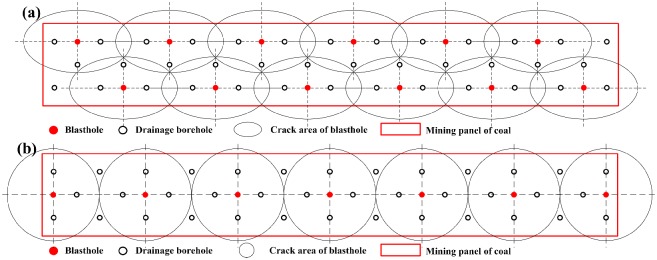
Blasthole layout for a mining panel (a) Design of in this paper (b) Conventional design.

Here, the exists gas overflow at working face during the mining process at No. 16 mining area of Second-1 coal seam in Jiulishan coal mine. The effect of gas extraction is so poor that it could not achieve the purpose of eliminating outburst, so it needs to increase permeability using blasting method. Two decent panel dimensions at a working face (7.1×50 meter, coal thickness is 7.1 meter and panel length is 50 meter) are used to design the blasthole layout for gas drainage, one is used with the method in this paper (Here it is called a new method), the other is used with the conventional method. As mentioned earlier, to ensure enhancing the effect of gas extraction and the outburst risk of coal seams is eliminated, panel area must be fully cracked using blastholes. Fig 13a shows the new method of the blasthole and gas drainage holes layout within the mining panel. Under this circumstance, the whole panel is fully covered by the crack area of each blasthole, and there is no gap area among blasholes in the panel. And Fig 13b shows the conventional method of the blasthole and gas drainage holes layout in near panel with the same size, which considers that the cracking area is a circle with the radius 4.0m. In these two decent panels (7.1×50 meter), the layout way of the blasthole and gas drainage hole is shown in Table 4, and these two methods have the same layout of gas drainage hole (The number of gas drainage holes is 38).

**Table 4:** The layout way of parameter.

Parameters	Blasthole	Gas drainage hole
Hole diameter (mm)	90	94
Hole depth (m)	60	65
Hole-sealed length (m)	10	12
Charge quantity (kg)	38.5	——
Negative pressure (kPa) for gas drainage	——	18.4

**Fig 14:**
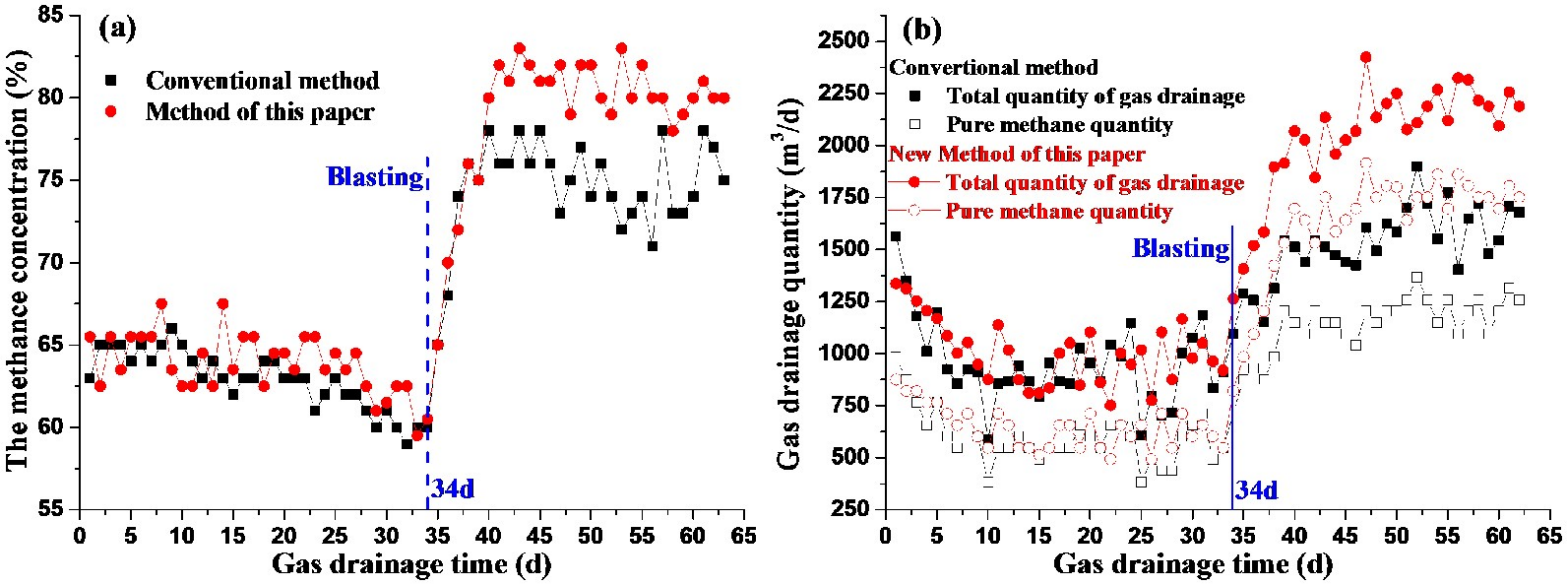
The effect of gas drainage before and after blasting with the two methods (a) The methane concentration (b) Gas drainage quantity.

In order to investigate the effect of pre-splitting blasting to improve gas drainage, continuous observation of these two areas before and after blasting is carried out for more than 60 days. And the data obtained are collated to compare the effect of gas drainage before and after deep-hole pre-splitting blasting with the two methods of blasthole layout in Fig 14. It can be seen from Fig 4 that not only the methane concentration but also gas drainage quantity of these two methods is consistent before deep-hole pre-splitting blasting. For example, the mean values of the methane concentration of conventional method and new method are 62.85% and 63.82% respectively, and the mean values of total quantity of gas drainage are 954.97m^3^/d and 1012.18m^3^/d respectively. On the 34^th^ day during gas drainage, deep-hole pre-splitting blasting was carried out. And after blasting, the methane concentration, total quantity and pure methane quantity of gas drainage all increase rapidly, and then stabilizes in the following period of time. However, comparing the conventional method with the new method of blasthole layout, the methane concentration, total quantity and pure methane quantity of gas drainage all increase significantly after blasting. For example, the mean values of above 3 type of measured data with new method are 79.14%, 2151.96 m^3^/d and 1736.52 m^3^/d, which are respectively 1.06, 1.36 and 1.46 times bigger than mean values of 74.59%, 1586.3 m^3^/d and 1189.57 m^3^/d with conventional method. Thus, the new method of blasthole layout has remarkable effect on gas drainage, so it is necessary to consider the coal seam anisotropy of deep-hole pre-splitting blasting for gas drainage.

## 5 Conclusion

Deep-hole pre-splitting blasting technology is applied widely to enhance gas drainage in low permeability and high gassy seam, and the effective crack distance of blasting is the key for blasthole layout. Considering the structural anisotropic characteristics of coal seam, the mechanical parameters of coal samples in different coring directions are obtained by the tensile and compressive tests. And the calculation results of the blasting crack distances are good agreement with the field test results. So according to the blasting crack distances in different directions, a new method of blasthhole layout in anisotropic coal seam is designed and applied, the gas drainage effect is investigated underground in-situ measurement.

1. From parallel bedding direction to perpendicular bedding direction, both compressive strength and tensile strength increase, and the compressive strength is about 13 times bigger than the tensile strength on the same direction.
2. The blasting crack distances increase in different directions with the tensile strength decreasing, and the distance in the direction of parallel to bedding is much bigger than that perpendicular bedding.
3. The blasting crack distances of calculation results are 2.615m and 4.511m in parallel and perpendicular bedding direction respectively, which are fine in agreement with the measured results between 2.5m and 3.0m in parallel bedding direction and between 4.0m and 5.0m in perpendicular bedding direction.
4. According to the calculation results of the blasting crack distances in different directions, the blasting crack zones can be approximately treated as an ellipse.
5. After blasting, the blasting crack effect on new method has greatly enhanced the methane concentration and gas drainage quantity underground in-situ measurement. And pure methane quantity with new method is about 1.46 times as large as that with conventional method.
